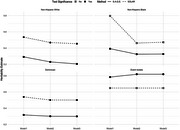# Dissecting Alzheimer Disease Heritability Across Multiple Populations

**DOI:** 10.1002/alz70855_102132

**Published:** 2025-12-23

**Authors:** Shiying Liu, William S Bush, Brian W Kunkle, Goldie S Byrd, Christiane Reitz, Giuseppe Tosto, Jeffery M Vance, Margaret Pericak‐Vance, Jonathan L Haines, Dana C. Crawford, Scott M Williams

**Affiliations:** ^1^ Department of Population and Quantitative Health Sciences, Case Western Reserve University, Cleveland, OH, USA; ^2^ Department of Population and Quantitative Health Sciences, Case Western Reserve University School of Medicine, Cleveland, OH, USA; ^3^ Dr. John T. Macdonald Foundation Department of Human Genetics, University of Miami Miller School of Medicine, Miami, FL, USA; ^4^ Wake Forest University School of Medicine, Winston‐Salem, NC, USA; ^5^ Columbia University, New York, NY, USA; ^6^ Case Western Reserve University School of Medicine, Cleveland, OH, USA

## Abstract

**Background:**

Late‐onset Alzheimer disease (LOAD) is highly heritable. Twin studies estimated up to 80% heritability in European populations. However, the range of narrow sense LOAD heritability estimates across multiple populations remains poorly understood, as does the relative contribution of factors, such as *APOE*, recorded race/ethnicity, and cohort effects.

**Method:**

We computed family‐based heritability leveraging Alzheimer's Disease Sequencing Project (ADSP) pedigrees and genome‐wide data across five distinct groups identified by recorded race/ethnicity: non‐Hispanic White (*n* = 7,024; 404 pedigrees), non‐Hispanic Black (*n* = 286; 13 pedigrees), Dominican (*n* = 3,988; 101 pedigrees), Puerto Rican (*n* = 132; 3 pedigrees), and Dutch Isolate (*n* = 655; 10 pedigrees). Family‐based heritability was estimated adjusting for 1) age and sex, 2) age, sex, and APOE ε4 carrier status, 3) age, sex, APOE ε4 carrier status, and cohort, using both Statistical Analysis for Genetic Epidemiology (S.A.G.E.) and Sequential Oligogenic Linkage Analysis Routines (SOLAR).

**Result:**

Regardless of method or adjustment, the resulting heritability estimates were highly variable across populations. Using S.A.G.E. adjusted for age and sex (model 1), estimates were highest for the Dutch isolates (78.3%) followed by non‐Hispanic Blacks (39.1%), Dominicans (31.7%), and non‐Hispanic Whites (29.1%). The inclusion of APOE ε4 carrier status (model 2) reduced heritability estimates by an average of 4.9%, with the largest reductions observed in non‐Hispanic Blacks (6.7%) and non‐Hispanic Whites (6.2%). Cohort adjustment (models 3) primarily affected the estimates for the non‐Hispanic Whites (2.4% decrease), reflecting their greater contributions to cohort diversity. The Puerto Rican dataset failed to converge in all models due to small sample size. In general, narrow sense heritability estimates were almost always higher (45.4% ‐ 80.0%) using SOLAR compared with S.A.G.E. (20.4% ‐ 81.9%), reflecting differences in modeling and handling of missing data, while relative patterns and trends remained consistent regardless of magnitude.

**Conclusion:**

While family‐based estimates complement previous twin‐based and genome‐wide studies, the wide range of narrow sense heritability estimated across populations reflects substantial variability in population‐specific and unmeasured extrinsic factors, such as lifestyle and behaviors, environmental exposures, and social determinants of health. Work supported by AG074865 and AG072547 is ongoing to estimate SNP‐based heritability in overlapping participants for direct comparisons with family‐based heritability.